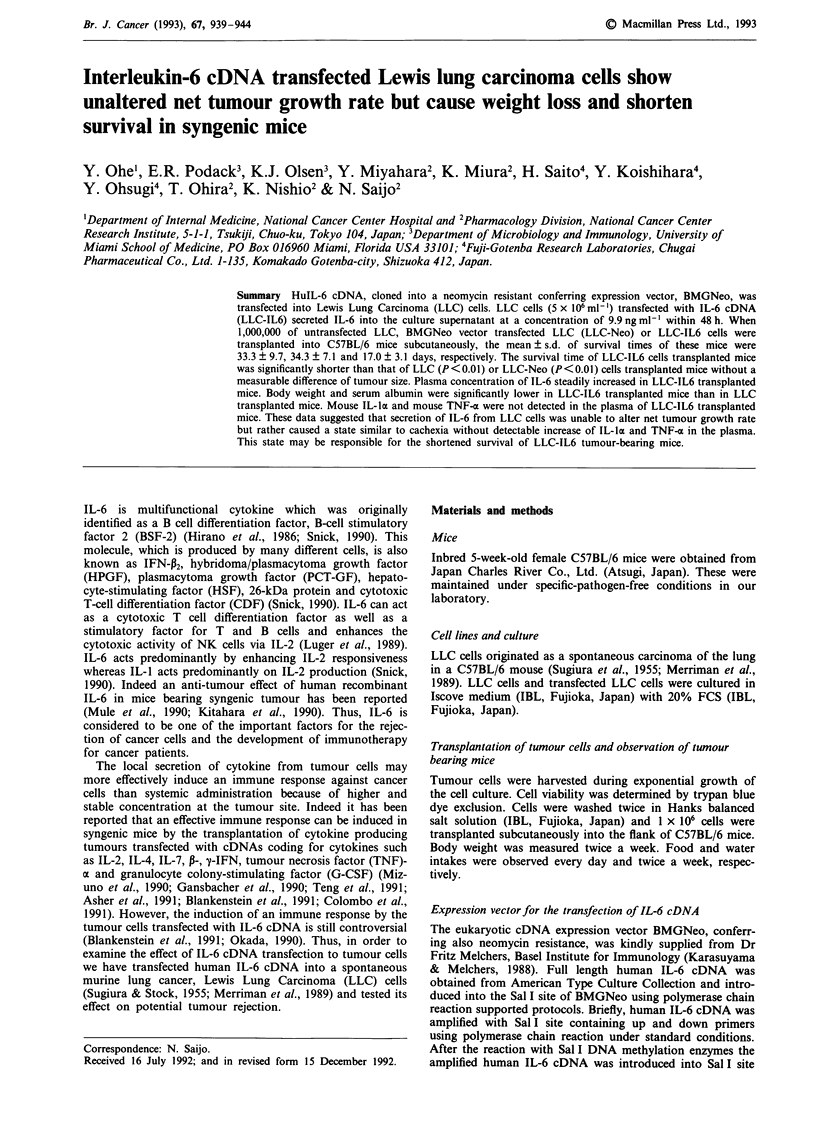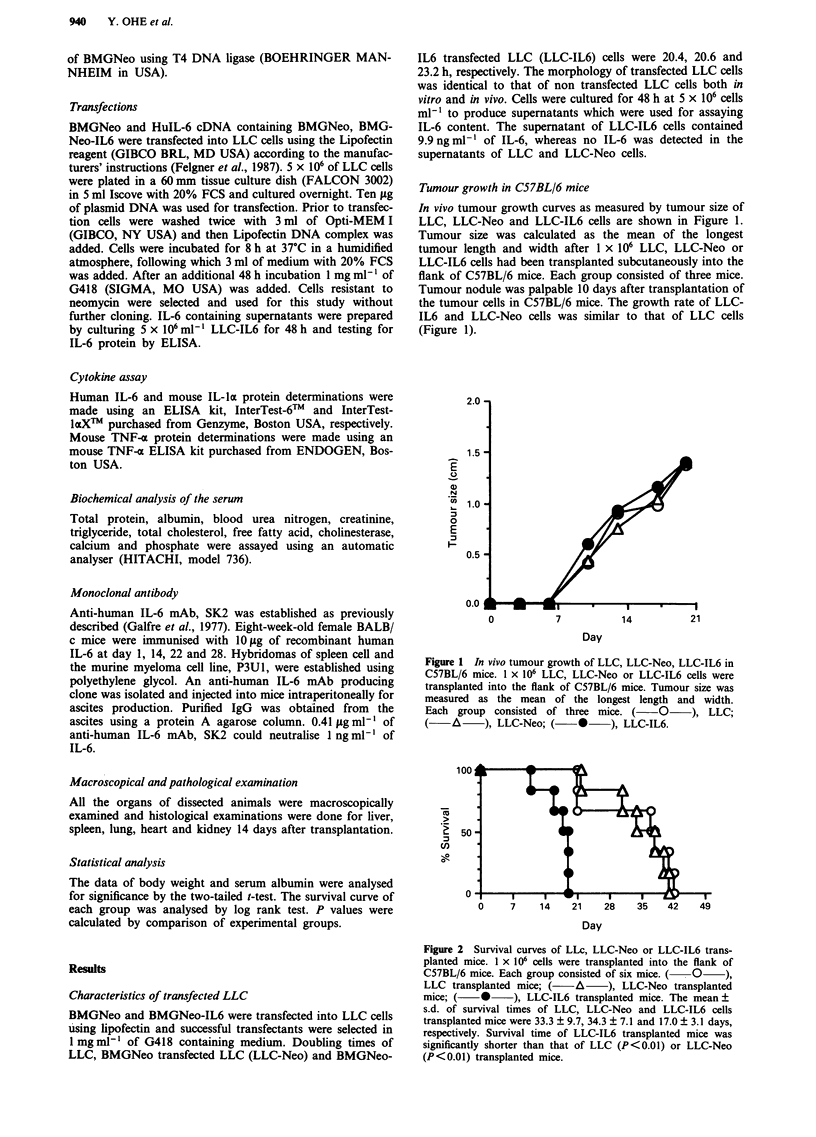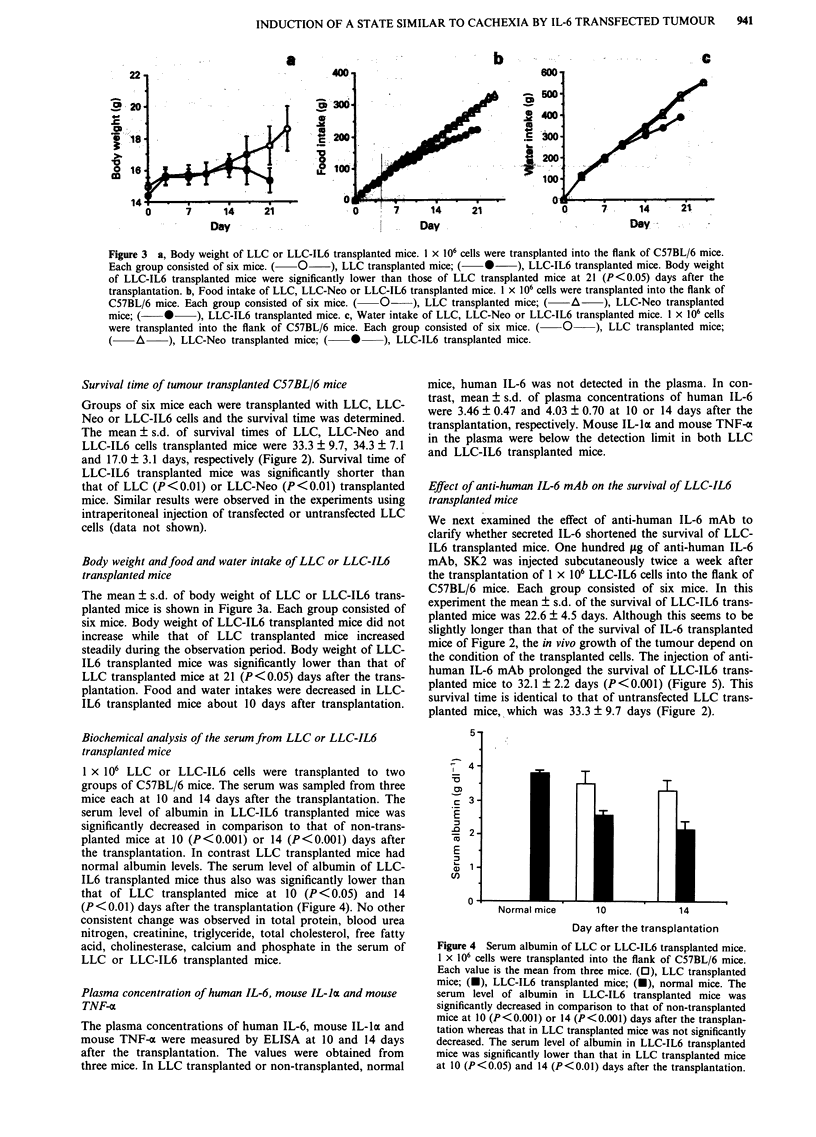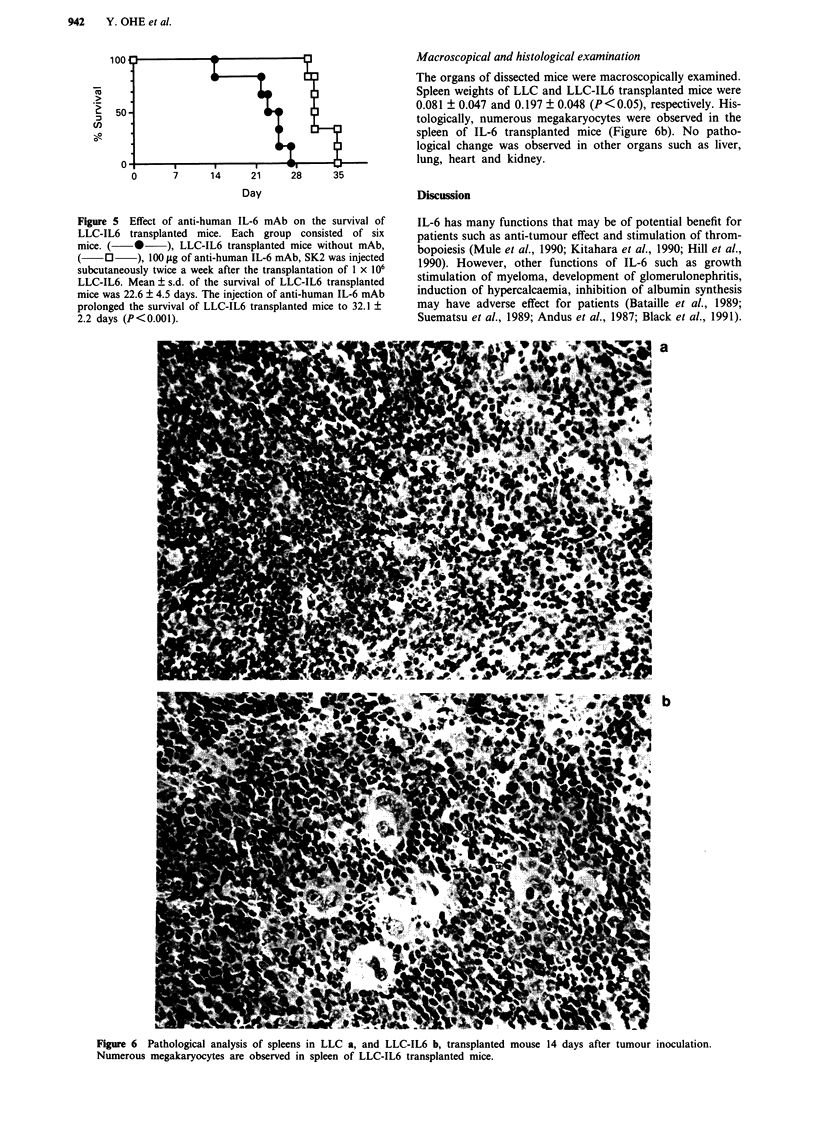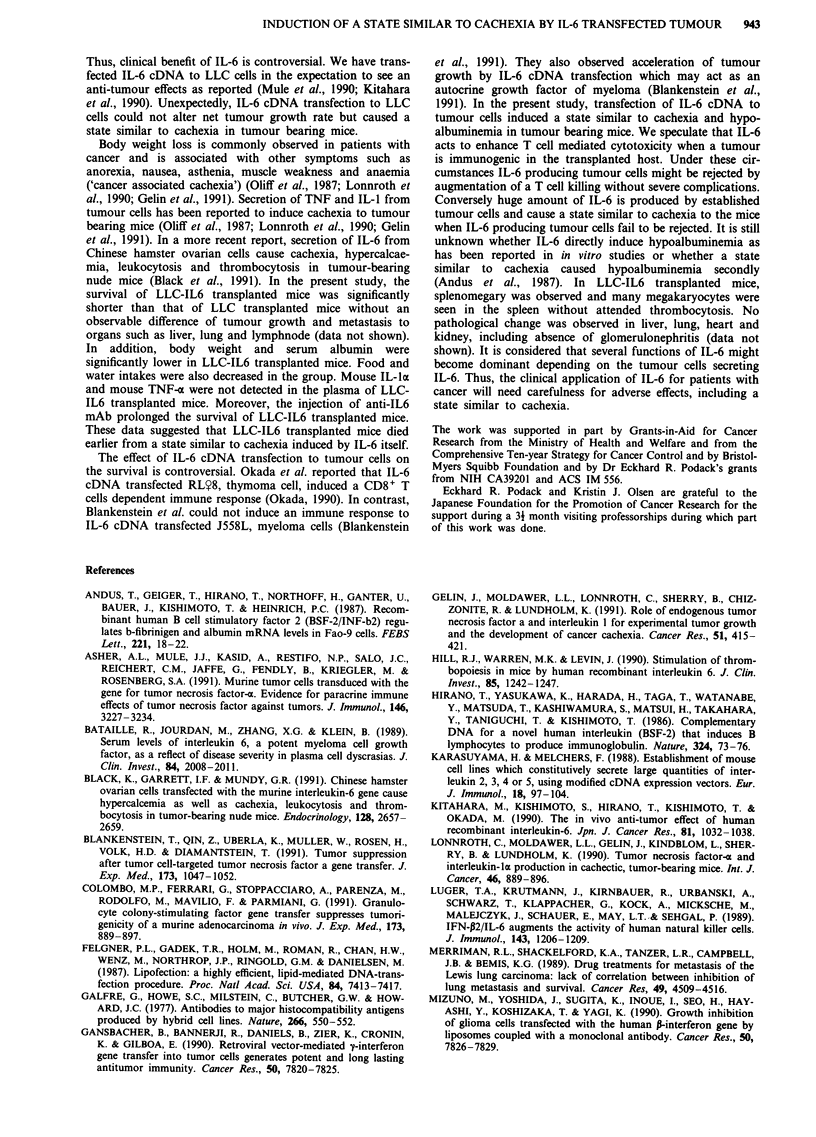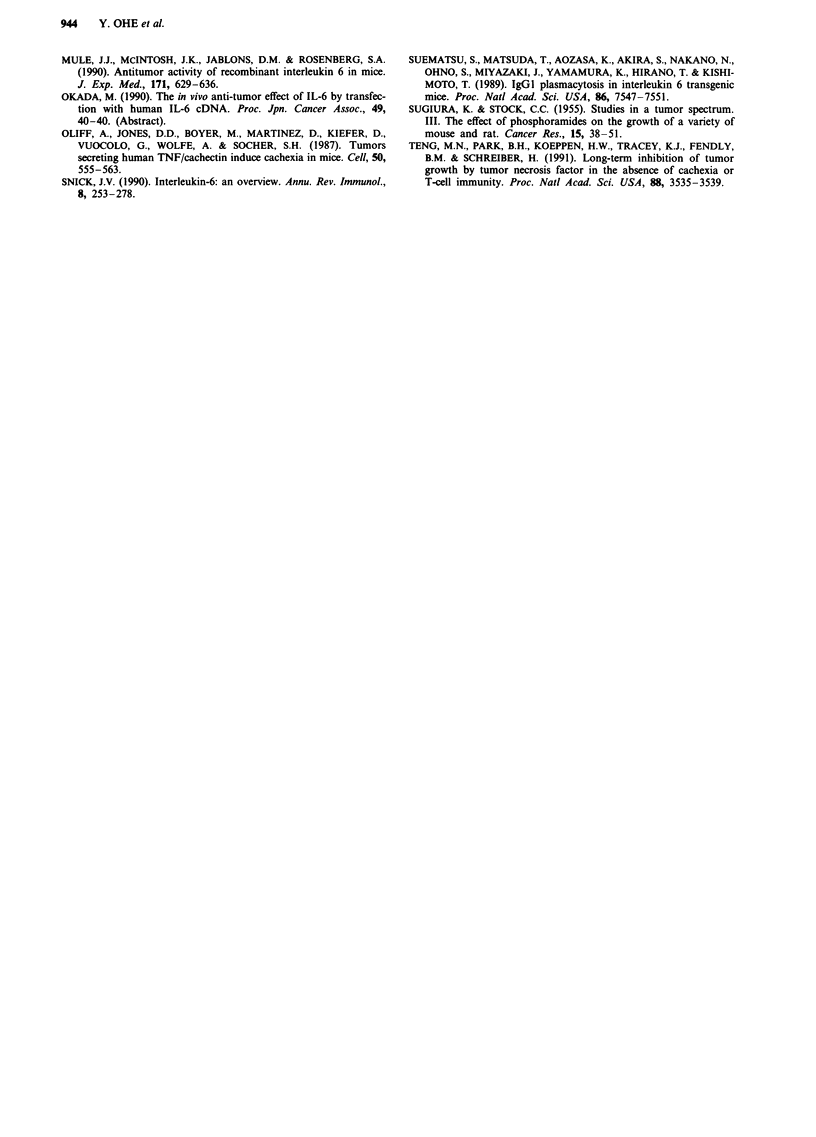# Interleukin-6 cDNA transfected Lewis lung carcinoma cells show unaltered net tumour growth rate but cause weight loss and shortened survival in syngeneic mice.

**DOI:** 10.1038/bjc.1993.174

**Published:** 1993-05

**Authors:** Y. Ohe, E. R. Podack, K. J. Olsen, Y. Miyahara, K. Miura, H. Saito, Y. Koishihara, Y. Ohsugi, T. Ohira, K. Nishio

**Affiliations:** Department of Internal Medicine, National Cancer Center Hospital, Tokyo, Japan.

## Abstract

**Images:**


Br. J. Cancer (1993), 67, 939-944 © Macmillan Press Ltd., 1993

### Interleukin-6 cDNA transfected Lewis lung carcinoma cells show unaltered net tumour growth rate but cause weight loss and shorten survival in syngenic mice

Y. Ohe[1], E.R. Podack[3], K.J. Olsen[3], Y. Miyahara[2], K. Miura[2], H. Saito[4], Y. Koishihara[4], Y. Ohsugi[4], T. Ohira[2], K. Nishio[2] & N. Saijo[2]

[1]*Department of Internal Medicine, National Cancer Center Hospital and* [2]*Pharmacology Division, National Cancer Center Research Institute, 5-1-1, Tsukiji, Chuo-ku, Tokyo 104, Japan;* [3]*Department of Microbiology and Immunology, University of Miami School of Medicine, PO Box 016960 Miami, Florida USA 33101;* [4]*Fuji-Gotenba Research Laboratories, Chugai Pharmaceutical Co., Ltd. 1-135, Komakado Gotenba-city, Shizuoka 412, Japan.*

**Summary** HuIL-6 cDNA, cloned into a neomycin resistant conferring expression vector, BMGNeo, was transfected into Lewis Lung Carcinoma (LLC) cells. LLC cells ($5 \times 10^6$ ml$^{-1}$) transfected with IL-6 cDNA (LLC-IL6) secreted IL-6 into the culture supernatant at a concentration of 9.9 ng ml$^{-1}$ within 48 h. When 1,000,000 of untransfected LLC, BMGNeo vector transfected LLC (LLC-Neo) or LLC-IL6 cells were transplanted into C57BL/6 mice subcutaneously, the mean ± s.d. of survival times of these mice were 33.3 ± 9.7, 34.3 ± 7.1 and 17.0 ± 3.1 days, respectively. The survival time of LLC-IL6 cells transplanted mice was significantly shorter than that of LLC ($P < 0.01$) or LLC-Neo ($P < 0.01$) cells transplanted mice without a measurable difference of tumour size. Plasma concentration of IL-6 steadily increased in LLC-IL6 transplanted mice. Body weight and serum albumin were significantly lower in LLC-IL6 transplanted mice than in LLC transplanted mice. Mouse IL-1α and mouse TNF-α were not detected in the plasma of LLC-IL6 transplanted mice. These data suggested that secretion of IL-6 from LLC cells was unable to alter net tumour growth rate but rather caused a state similar to cachexia without detectable increase of IL-1α and TNF-α in the plasma. This state may be responsible for the shortened survival of LLC-IL6 tumour-bearing mice.

IL-6 is multifunctional cytokine which was originally identified as a B cell differentiation factor, B-cell stimulatory factor 2 (BSF-2) (Hirano *et al.*, 1986; Snick, 1990). This molecule, which is produced by many different cells, is also known as IFN-$\beta_2$, hybridoma/plasmacytoma growth factor (HPGF), plasmacytoma growth factor (PCT-GF), hepatocyte-stimulating factor (HSF), 26-kDa protein and cytotoxic T-cell differentiation factor (CDF) (Snick, 1990). IL-6 can act as a cytotoxic T cell differentiation factor as well as a stimulatory factor for T and B cells and enhances the cytotoxic activity of NK cells via IL-2 (Luger *et al.*, 1989). IL-6 acts predominantly by enhancing IL-2 responsiveness whereas IL-1 acts predominantly on IL-2 production (Snick, 1990). Indeed an anti-tumour effect of human recombinant IL-6 in mice bearing syngenic tumour has been reported (Mule *et al.*, 1990; Kitahara *et al.*, 1990). Thus, IL-6 is considered to be one of the important factors for the rejection of cancer cells and the development of immunotherapy for cancer patients.

The local secretion of cytokine from tumour cells may more effectively induce an immune response against cancer cells than systemic administration because of higher and stable concentration at the tumour site. Indeed it has been reported that an effective immune response can be induced in syngenic mice by the transplantation of cytokine producing tumours transfected with cDNAs coding for cytokines such as IL-2, IL-4, IL-7, β-, γ-IFN, tumour necrosis factor (TNF)-α and granulocyte colony-stimulating factor (G-CSF) (Mizuno *et al.*, 1990; Gansbacher *et al.*, 1990; Teng *et al.*, 1991; Asher *et al.*, 1991; Blankenstein *et al.*, 1991; Colombo *et al.*, 1991). However, the induction of an immune response by the tumour cells transfected with IL-6 cDNA is still controversial (Blankenstein *et al.*, 1991; Okada, 1990). Thus, in order to examine the effect of IL-6 cDNA transfection to tumour cells we have transfected human IL-6 cDNA into a spontaneous murine lung cancer, Lewis Lung Carcinoma (LLC) cells (Sugiura & Stock, 1955; Merriman *et al.*, 1989) and tested its effect on potential tumour rejection.

Correspondence: N. Saijo.
Received 16 July 1992; and in revised form 15 December 1992.

### Materials and methods

### Mice

Inbred 5-week-old female C57BL/6 mice were obtained from Japan Charles River Co., Ltd. (Atsugi, Japan). These were maintained under specific-pathogen-free conditions in our laboratory.

### Cell lines and culture

LLC cells originated as a spontaneous carcinoma of the lung in a C57BL/6 mouse (Sugiura *et al.*, 1955; Merriman *et al.*, 1989). LLC cells and transfected LLC cells were cultured in Iscove medium (IBL, Fujioka, Japan) with 20% FCS (IBL, Fujioka, Japan).

### Transplantation of tumour cells and observation of tumour bearing mice

Tumour cells were harvested during exponential growth of the cell culture. Cell viability was determined by trypan blue dye exclusion. Cells were washed twice in Hanks balanced salt solution (IBL, Fujioka, Japan) and $1 \times 10^6$ cells were transplanted subcutaneously into the flank of C57BL/6 mice. Body weight was measured twice a week. Food and water intakes were observed every day and twice a week, respectively.

### Expression vector for the transfection of IL-6 cDNA

The eukaryotic cDNA expression vector BMGNeo, conferring also neomycin resistance, was kindly supplied from Dr Fritz Melchers, Basel Institute for Immunology (Karasuyama & Melchers, 1988). Full length human IL-6 cDNA was obtained from American Type Culture Collection and introduced into the Sal I site of BMGNeo using polymerase chain reaction supported protocols. Briefly, human IL-6 cDNA was amplified with Sal I site containing up and down primers using polymerase chain reaction under standard conditions. After the reaction with Sal I DNA methylation enzymes the amplified human IL-6 cDNA was introduced into Sal I site of BMGNeo using T4 DNA ligase (BOEHRINGER MANNHEIM in USA).

*Transfections*

BMGNeo and HuIL-6 cDNA containing BMGNeo, BMG-Neo-IL6 were transfected into LLC cells using the Lipofectin reagent (GIBCO BRL, MD USA) according to the manufacturers' instructions (Felgner *et al.*, 1987). 5 × 10^6 of LLC cells were plated in a 60 mm tissue culture dish (FALCON 3002) in 5 ml Iscove with 20% FCS and cultured overnight. Ten μg of plasmid DNA was used for transfection. Prior to transfection cells were washed twice with 3 ml of Opti-MEM I (GIBCO, NY USA) and then Lipofectin DNA complex was added. Cells were incubated for 8 h at 37°C in a humidified atmosphere, following which 3 ml of medium with 20% FCS was added. After an additional 48 h incubation 1 mg $ml^{-1}$ of G418 (SIGMA, MO USA) was added. Cells resistant to neomycin were selected and used for this study without further cloning. IL-6 containing supernatants were prepared by culturing 5 × $10^6$ $ml^{-1}$ LLC-IL6 for 48 h and testing for IL-6 protein by ELISA.

*Cytokine assay*

Human IL-6 and mouse IL-1α protein determinations were made using an ELISA kit, InterTest-6™ and InterTest-1αX™ purchased from Genzyme, Boston USA, respectively. Mouse TNF-α protein determinations were made using an mouse TNF-α ELISA kit purchased from ENDOGEN, Boston USA.

*Biochemical analysis of the serum*

Total protein, albumin, blood urea nitrogen, creatinine, triglyceride, total cholesterol, free fatty acid, cholinesterase, calcium and phosphate were assayed using an automatic analyser (HITACHI, model 736).

*Monoclonal antibody*

Anti-human IL-6 mAb, SK2 was established as previously described (Galfre *et al.*, 1977). Eight-week-old female BALB/c mice were immunised with 10 μg of recombinant human IL-6 at day 1, 14, 22 and 28. Hybridomas of spleen cell and the murine myeloma cell line, P3U1, were established using polyethylene glycol. An anti-human IL-6 mAb producing clone was isolated and injected into mice intraperitoneally for ascites production. Purified IgG was obtained from the ascites using a protein A agarose column. 0.41 μg $ml^{-1}$ of anti-human IL-6 mAb, SK2 could neutralise 1 ng $ml^{-1}$ of IL-6.

*Macroscopical and pathological examination*

All the organs of dissected animals were macroscopically examined and histological examinations were done for liver, spleen, lung, heart and kidney 14 days after transplantation.

*Statistical analysis*

The data of body weight and serum albumin were analysed for significance by the two-tailed *t*-test. The survival curve of each group was analysed by log rank test. *P* values were calculated by comparison of experimental groups.

### Results

*Characteristics of transfected LLC*

BMGNeo and BMGNeo-IL6 were transfected into LLC cells using lipofectin and successful transfectants were selected in 1 mg $ml^{-1}$ of G418 containing medium. Doubling times of LLC, BMGNeo transfected LLC (LLC-Neo) and BMGNeo-IL6 transfected LLC (LLC-IL6) cells were 20.4, 20.6 and 23.2 h, respectively. The morphology of transfected LLC cells was identical to that of non transfected LLC cells both *in vitro* and *in vivo*. Cells were cultured for 48 h at 5 × $10^6$ cells $ml^{-1}$ to produce supernatants which were used for assaying IL-6 content. The supernatant of LLC-IL6 cells contained 9.9 ng $ml^{-1}$ of IL-6, whereas no IL-6 was detected in the supernatants of LLC and LLC-Neo cells.

*Tumour growth in C57BL/6 mice*

*In vivo* tumour growth curves as measured by tumour size of LLC, LLC-Neo and LLC-IL6 cells are shown in Figure 1. Tumour size was calculated as the mean of the longest tumour length and width after 1 × $10^6$ LLC, LLC-Neo or LLC-IL6 cells had been transplanted subcutaneously into the flank of C57BL/6 mice. Each group consisted of three mice. Tumour nodule was palpable 10 days after transplantation of the tumour cells in C57BL/6 mice. The growth rate of LLC-IL6 and LLC-Neo cells was similar to that of LLC cells (Figure 1).

**Figure 1** *In vivo* tumour growth of LLC, LLC-Neo, LLC-IL6 in C57BL/6 mice. 1 × $10^6$ LLC, LLC-Neo or LLC-IL6 cells were transplanted into the flank of C57BL/6 mice. Tumour size was measured as the mean of the longest length and width. Each group consisted of three mice. (——O——), LLC; (——Δ——), LLC-Neo; (——●——), LLC-IL6.

**Figure 2** Survival curves of LLc, LLC-Neo or LLC-IL6 transplanted mice. 1 × $10^6$ cells were transplanted into the flank of C57BL/6 mice. Each group consisted of six mice. (——O——), LLC transplanted mice; (——Δ——), LLC-Neo transplanted mice; (——●——), LLC-IL6 transplanted mice. The mean ± s.d. of survival times of LLC, LLC-Neo and LLC-IL6 cells transplanted mice were 33.3 ± 9.7, 34.3 ± 7.1 and 17.0 ± 3.1 days, respectively. Survival time of LLC-IL6 transplanted mice was significantly shorter than that of LLC ($P < 0.01$) or LLC-Neo ($P < 0.01$) transplanted mice.

**Figure 3** **a**, Body weight of LLC or LLC-IL6 transplanted mice. 1 × $10^6$ cells were transplanted into the flank of C57BL/6 mice. Each group consisted of six mice. (——O——), LLC transplanted mice; (——●——), LLC-IL6 transplanted mice. Body weight of LLC-IL6 transplanted mice were significantly lower than those of LLC transplanted mice at 21 ($P<0.05$) days after the transplantation. **b**, Food intake of LLC, LLC-Neo or LLC-IL6 transplanted mice. 1 × $10^6$ cells were transplanted into the flank of C57BL/6 mice. Each group consisted of six mice. (——O——), LLC transplanted mice; (——Δ——), LLC-Neo transplanted mice; (——●——), LLC-IL6 transplanted mice. **c**, Water intake of LLC, LLC-Neo or LLC-IL6 transplanted mice. 1 × $10^6$ cells were transplanted into the flank of C57BL/6 mice. Each group consisted of six mice. (——O——), LLC transplanted mice; (——Δ——), LLC-Neo transplanted mice; (——●——), LLC-IL6 transplanted mice.

*Survival time of tumour transplanted C57BL/6 mice*

Groups of six mice each were transplanted with LLC, LLC-Neo or LLC-IL6 cells and the survival time was determined. The mean ± s.d. of survival times of LLC, LLC-Neo and LLC-IL6 cells transplanted mice were 33.3 ± 9.7, 34.3 ± 7.1 and 17.0 ± 3.1 days, respectively (Figure 2). Survival time of LLC-IL6 transplanted mice was significantly shorter than that of LLC ($P<0.01$) or LLC-Neo ($P<0.01$) transplanted mice. Similar results were observed in the experiments using intraperitoneal injection of transfected or untransfected LLC cells (data not shown).

*Body weight and food and water intake of LLC or LLC-IL6 transplanted mice*

The mean ± s.d. of body weight of LLC or LLC-IL6 transplanted mice is shown in Figure 3a. Each group consisted of six mice. Body weight of LLC-IL6 transplanted mice did not increase while that of LLC transplanted mice increased steadily during the observation period. Body weight of LLC-IL6 transplanted mice was significantly lower than that of LLC transplanted mice at 21 ($P<0.05$) days after the transplantation. Food and water intakes were decreased in LLC-IL6 transplanted mice about 10 days after transplantation.

*Biochemical analysis of the serum from LLC or LLC-IL6 transplanted mice*

1 × $10^6$ LLC or LLC-IL6 cells were transplanted to two groups of C57BL/6 mice. The serum was sampled from three mice each at 10 and 14 days after the transplantation. The serum level of albumin in LLC-IL6 transplanted mice was significantly decreased in comparison to that of non-transplanted mice at 10 ($P<0.001$) or 14 ($P<0.001$) days after the transplantation. In contrast LLC transplanted mice had normal albumin levels. The serum level of albumin of LLC-IL6 transplanted mice thus also was significantly lower than that of LLC transplanted mice at 10 ($P<0.05$) and 14 ($P<0.01$) days after the transplantation (Figure 4). No other consistent change was observed in total protein, blood urea nitrogen, creatinine, triglyceride, total cholesterol, free fatty acid, cholinesterase, calcium and phosphate in the serum of LLC or LLC-IL6 transplanted mice.

*Plasma concentration of human IL-6, mouse IL-1α and mouse TNF-α*

The plasma concentrations of human IL-6, mouse IL-1α and mouse TNF-α were measured by ELISA at 10 and 14 days after the transplantation. The values were obtained from three mice. In LLC transplanted or non-transplanted, normal mice, human IL-6 was not detected in the plasma. In contrast, mean ± s.d. of plasma concentrations of human IL-6 were 3.46 ± 0.47 and 4.03 ± 0.70 at 10 or 14 days after the transplantation, respectively. Mouse IL-1α and mouse TNF-α in the plasma were below the detection limit in both LLC and LLC-IL6 transplanted mice.

*Effect of anti-human IL-6 mAb on the survival of LLC-IL6 transplanted mice*

We next examined the effect of anti-human IL-6 mAb to clarify whether secreted IL-6 shortened the survival of LLC-IL6 transplanted mice. One hundred μg of anti-human IL-6 mAb, SK2 was injected subcutaneously twice a week after the transplantation of 1 × $10^6$ LLC-IL6 cells into the flank of C57BL/6 mice. Each group consisted of six mice. In this experiment the mean ± s.d. of the survival of LLC-IL6 transplanted mice was 22.6 ± 4.5 days. Although this seems to be slightly longer than that of the survival of IL-6 transplanted mice of Figure 2, the *in vivo* growth of the tumour depend on the condition of the transplanted cells. The injection of anti-human IL-6 mAb prolonged the survival of LLC-IL6 transplanted mice to 32.1 ± 2.2 days ($P<0.001$) (Figure 5). This survival time is identical to that of untransfected LLC transplanted mice, which was 33.3 ± 9.7 days (Figure 2).

**Figure 4** Serum albumin of LLC or LLC-IL6 transplanted mice. 1 × $10^6$ cells were transplanted into the flank of C57BL/6 mice. Each value is the mean from three mice. (□), LLC transplanted mice; (■), LLC-IL6 transplanted mice; (■), normal mice. The serum level of albumin in LLC-IL6 transplanted mice was significantly decreased in comparison to that of non-transplanted mice at 10 ($P<0.001$) or 14 ($P<0.001$) days after the transplantation whereas that in LLC transplanted mice was not significantly decreased. The serum level of albumin in LLC-IL6 transplanted mice was significantly lower than that in LLC transplanted mice at 10 ($P<0.05$) and 14 ($P<0.01$) days after the transplantation.

**Figure 5** Effect of anti-human IL-6 mAb on the survival of LLC-IL6 transplanted mice. Each group consisted of six mice. (——●——), LLC-IL6 transplanted mice without mAb, (——□——), 100 μg of anti-human IL-6 mAb, SK2 was injected subcutaneously twice a week after the transplantation of 1 × $10^6$ LLC-IL6. Mean ± s.d. of the survival of LLC-IL6 transplanted mice was 22.6 ± 4.5 days. The injection of anti-human IL-6 mAb prolonged the survival of LLC-IL6 transplanted mice to 32.1 ± 2.2 days ($P<0.001$).

*Macroscopical and histological examination*

The organs of dissected mice were macroscopically examined. Spleen weights of LLC and LLC-IL6 transplanted mice were 0.081 ± 0.047 and 0.197 ± 0.048 ($P<0.05$), respectively. Histologically, numerous megakaryocytes were observed in the spleen of IL-6 transplanted mice (Figure 6b). No pathological change was observed in other organs such as liver, lung, heart and kidney.

### Discussion

IL-6 has many functions that may be of potential benefit for patients such as anti-tumour effect and stimulation of thrombopoiesis (Mule *et al.*, 1990; Kitahara *et al.*, 1990; Hill *et al.*, 1990). However, other functions of IL-6 such as growth stimulation of myeloma, development of glomerulonephritis, induction of hypercalcaemia, inhibition of albumin synthesis may have adverse effect for patients (Bataille *et al.*, 1989; Suematsu *et al.*, 1989; Andus *et al.*, 1987; Black *et al.*, 1991).

**Figure 6** Pathological analysis of spleens in LLC **a**, and LLC-IL6 **b**, transplanted mouse 14 days after tumour inoculation. Numerous megakaryocytes are observed in spleen of LLC-IL6 transplanted mice.

Thus, clinical benefit of IL-6 is controversial. We have transfected IL-6 cDNA to LLC cells in the expectation to see an anti-tumour effects as reported (Mule *et al.*, 1990; Kitahara *et al.*, 1990). Unexpectedly, IL-6 cDNA transfection to LLC cells could not alter net tumour growth rate but caused a state similar to cachexia in tumour bearing mice.

Body weight loss is commonly observed in patients with cancer and is associated with other symptoms such as anorexia, nausea, asthenia, muscle weakness and anaemia ('cancer associated cachexia') (Oliff *et al.*, 1987; Lonnroth *et al.*, 1990; Gelin *et al.*, 1991). Secretion of TNF and IL-1 from tumour cells has been reported to induce cachexia to tumour bearing mice (Oliff *et al.*, 1987; Lonnroth *et al.*, 1990; Gelin *et al.*, 1991). In a more recent report, secretion of IL-6 from Chinese hamster ovarian cells cause cachexia, hypercalcaemia, leukocytosis and thrombocytosis in tumour-bearing nude mice (Black *et al.*, 1991). In the present study, the survival of LLC-IL6 transplanted mice was significantly shorter than that of LLC transplanted mice without an observable difference of tumour growth and metastasis to organs such as liver, lung and lymphnode (data not shown). In addition, body weight and serum albumin were significantly lower in LLC-IL6 transplanted mice. Food and water intakes were also decreased in the group. Mouse IL-1α and mouse TNF-α were not detected in the plasma of LLC-IL6 transplanted mice. Moreover, the injection of anti-IL6 mAb prolonged the survival of LLC-IL6 transplanted mice. These data suggested that LLC-IL6 transplanted mice died earlier from a state similar to cachexia induced by IL-6 itself.

The effect of IL-6 cDNA transfection to tumour cells on the survival is controversial. Okada *et al.* reported that IL-6 cDNA transfected RL♀8, thymoma cell, induced a $CD8^+$ T cells dependent immune response (Okada, 1990). In contrast, Blankenstein *et al.* could not induce an immune response to IL-6 cDNA transfected J558L, myeloma cells (Blankenstein *et al.*, 1991). They also observed acceleration of tumour growth by IL-6 cDNA transfection which may act as an autocrine growth factor of myeloma (Blankenstein *et al.*, 1991). In the present study, transfection of IL-6 cDNA to tumour cells induced a state similar to cachexia and hypoalbuminemia in tumour bearing mice. We speculate that IL-6 acts to enhance T cell mediated cytotoxicity when a tumour is immunogenic in the transplanted host. Under these circumstances IL-6 producing tumour cells might be rejected by augmentation of a T cell killing without severe complications. Conversely huge amount of IL-6 is produced by established tumour cells and cause a state similar to cachexia to the mice when IL-6 producing tumour cells fail to be rejected. It is still unknown whether IL-6 directly induce hypoalbuminemia as has been reported in *in vitro* studies or whether a state similar to cachexia caused hypoalbuminemia secondly (Andus *et al.*, 1987). In LLC-IL6 transplanted mice, splenomegary was observed and many megakaryocytes were seen in the spleen without attended thrombocytosis. No pathological change was observed in liver, lung, heart and kidney, including absence of glomerulonephritis (data not shown). It is considered that several functions of IL-6 might become dominant depending on the tumour cells secreting IL-6. Thus, the clinical application of IL-6 for patients with cancer will need carefulness for adverse effects, including a state similar to cachexia.

The work was supported in part by Grants-in-Aid for Cancer Research from the Ministry of Health and Welfare and from the Comprehensive Ten-year Strategy for Cancer Control and by Bristol-Myers Squibb Foundation and by Dr Eckhard R. Podack's grants from NIH CA39201 and ACS IM 556.

Eckhard R. Podack and Kristin J. Olsen are grateful to the Japanese Foundation for the Promotion of Cancer Research for the support during a 3½ month visiting professorships during which part of this work was done.

### References

ANDUS, T., GEIGER, T., HIRANO, T., NORTHOFF, H., GANTER, U., BAUER, J., KISHIMOTO, T. & HEINRICH, P.C. (1987). Recombinant human B cell stimulatory factor 2 (BSF-2/INF-b2) regulates b-fibrinigen and albumin mRNA levels in Fao-9 cells. *FEBS Lett.*, **221**, 18–22.

ASHER, A.L., MULE, J.J., KASID, A., RESTIFO, N.P., SALO, J.C., REICHERT, C.M., JAFFE, G., FENDLY, B., KRIEGLER, M. & ROSENBERG, S.A. (1991). Murine tumor cells transduced with the gene for tumor necrosis factor-α. Evidence for paracrine immune effects of tumor necrosis factor against tumors. *J. Immunol.*, **146**, 3227–3234.

BATAILLE, R., JOURDAN, M., ZHANG, X.G. & KLEIN, B. (1989). Serum levels of interleukin 6, a potent myeloma cell growth factor, as a reflect of disease severity in plasma cell dyscrasias. *J. Clin. Invest.*, **84**, 2008–2011.

BLACK, K., GARRETT, I.F. & MUNDY, G.R. (1991). Chinese hamster ovarian cells transfected with the murine interleukin-6 gene cause hypercalcemia as well as cachexia, leukocytosis and thrombocytosis in tumor-bearing nude mice. *Endocrinology*, **128**, 2657–2659.

BLANKENSTEIN, T., QIN, Z., UBERLA, K., MULLER, W., ROSEN, H., VOLK, H.D. & DIAMANTSTEIN, T. (1991). Tumor suppression after tumor cell-targeted tumor necrosis factor a gene transfer. *J. Exp. Med.*, **173**, 1047–1052.

COLOMBO, M.P., FERRARI, G., STOPPACCIARO, A., PARENZA, M., RODOLFO, M., MAVILIO, F. & PARMIANI, G. (1991). Granulocyte colony-stimulating factor gene transfer suppresses tumorigenicity of a murine adenocarcinoma *in vivo*. *J. Exp. Med.*, **173**, 889–897.

FELGNER, P.L., GADEK, T.R., HOLM, M., ROMAN, R., CHAN, H.W., WENZ, M., NORTHROP, J.P., RINGOLD, G.M. & DANIELSEN, M. (1987). Lipofection: a highly efficient, lipid-mediated DNA-transfection procedure. *Proc. Natl Acad. Sci. USA*, **84**, 7413–7417.

GALFRE, G., HOWE, S.C., MILSTEIN, C., BUTCHER, G.W. & HOWARD, J.C. (1977). Antibodies to major histocompatibility antigens produced by hybrid cell lines. *Nature*, **266**, 550–552.

GANSBACHER, B., BANNERJI, R., DANIELS, B., ZIER, K., CRONIN, K. & GILBOA, E. (1990). Retroviral vector-mediated γ-interferon gene transfer into tumor cells generates potent and long lasting antitumor immunity. *Cancer Res.*, **50**, 7820–7825.

GELIN, J., MOLDAWER, L.L., LONNROTH, C., SHERRY, B., CHIZZONITE, R. & LUNDHOLM, K. (1991). Role of endogenous tumor necrosis factor a and interleukin 1 for experimental tumor growth and the development of cancer cachexia. *Cancer Res.*, **51**, 415–421.

HILL, R.J., WARREN, M.K. & LEVIN, J. (1990). Stimulation of thrombopoiesis in mice by human recombinant interleukin 6. *J. Clin. Invest.*, **85**, 1242–1247.

HIRANO, T., YASUKAWA, K., HARADA, H., TAGA, T., WATANABE, Y., MATSUDA, T., KASHIWAMURA, S., MATSUI, H., TAKAHARA, Y., TANIGUCHI, T. & KISHIMOTO, T. (1986). Complementary DNA for a novel human interleukin (BSF-2) that induces B lymphocytes to produce immunoglobulin. *Nature*, **324**, 73–76.

KARASUYAMA, H. & MELCHERS, F. (1988). Establishment of mouse cell lines which constitutively secrete large quantities of interleukin 2, 3, 4 or 5, using modified cDNA expression vectors. *Eur. J. Immunol.*, **18**, 97–104.

KITAHARA, M., KISHIMOTO, S., HIRANO, T., KISHIMOTO, T. & OKADA, M. (1990). The in vivo anti-tumor effect of human recombinant interleukin-6. *Jpn. J. Cancer Res.*, **81**, 1032–1038.

LONNROTH, C., MOLDAWER, L.L., GELIN, J., KINDBLOM, L., SHERRY, B. & LUNDHOLM, K. (1990). Tumor necrosis factor-α and interleukin-1α production in cachectic, tumor-bearing mice. *Int. J. Cancer*, **46**, 889–896.

LUGER, T.A., KRUTMANN, J., KIRNBAUER, R., URBANSKI, A., SCHWARZ, T., KLAPPACHER, G., KOCK, A., MICKSCHE, M., MALEJCZYK, J., SCHAUER, E., MAY, L.T. & SEHGAL, P. (1989). IFN-β2/IL-6 augments the activity of human natural killer cells. *J. Immunol.*, **143**, 1206–1209.

MERRIMAN, R.L., SHACKELFORD, K.A., TANZER, L.R., CAMPBELL, J.B. & BEMIS, K.G. (1989). Drug treatments for metastasis of the Lewis lung carcinoma: lack of correlation between inhibition of lung metastasis and survival. *Cancer Res*, **49**, 4509–4516.

MIZUNO, M., YOSHIDA, J., SUGITA, K., INOUE, I., SEO, H., HAYASHI, Y., KOSHIZAKA, T. & YAGI, K. (1990). Growth inhibition of glioma cells transfected with the human β-interferon gene by liposomes coupled with a monoclonal antibody. *Cancer Res.*, **50**, 7826–7829.

MULE, J.J., McINTOSH, J.K., JABLONS, D.M. & ROSENBERG, S.A. (1990). Antitumor activity of recombinant interleukin 6 in mice. *J. Exp. Med.*, **171**, 629-636.

OKADA, M. (1990). The *in vivo* anti-tumor effect of IL-6 by transfection with human IL-6 cDNA. *Proc. Jpn. Cancer Assoc.*, **49**, 40-40. (Abstract).

OLIFF, A., JONES, D.D., BOYER, M., MARTINEZ, D., KIEFER, D., VUOCOLO, G., WOLFE, A. & SOCHER, S.H. (1987). Tumors secreting human TNF/cachectin induce cachexia in mice. *Cell*, **50**, 555-563.

SNICK, J.V. (1990). Interleukin-6: an overview. *Annu. Rev. Immunol.*, **8**, 253-278.

SUEMATSU, S., MATSUDA, T., AOZASA, K., AKIRA, S., NAKANO, N., OHNO, S., MIYAZAKI, J., YAMAMURA, K., HIRANO, T. & KISHIMOTO, T. (1989). IgG1 plasmacytosis in interleukin 6 transgenic mice. *Proc. Natl Acad. Sci. USA*, **86**, 7547-7551.

SUGIURA, K. & STOCK, C.C. (1955). Studies in a tumor spectrum. III. The effect of phosphoramides on the growth of a variety of mouse and rat. *Cancer Res.*, **15**, 38-51.

TENG, M.N., PARK, B.H., KOEPPEN, H.W., TRACEY, K.J., FENDLY, B.M. & SCHREIBER, H. (1991). Long-term inhibition of tumor growth by tumor necrosis factor in the absence of cachexia or T-cell immunity. *Proc. Natl Acad. Sci. USA*, **88**, 3535-3539.